# Gene expression profiling in PBMCs for acute rejection in lung transplant recipients reveals myeloid responses

**DOI:** 10.3389/frtra.2024.1508419

**Published:** 2024-12-18

**Authors:** Siqi Liu, Johanna Westra, Shixian Hu, Erik A. M. Verschuuren, Léon C. van Kempen, Debbie van Baarle, Nico A. Bos

**Affiliations:** ^1^ Department of Rheumatology and Clinical Immunology, University Medical Center Groningen, University of Groningen, Groningen, Netherlands; ^2^ Institute of Precision Medicine, The First Affiliated Hospital, Sun Yat-Sen University, Guangzhou, Guangdong, China; ^3^ Department of Pulmonary Diseases, University Medical Center Groningen, University of Groningen, Groningen, Netherlands; ^4^ Department of Pathology and Medical Biology, University Medical Center Groningen, University of Groningen, Groningen, Netherlands; ^5^ Department of Pathology, University of Antwerp, Antwerp University Hospital, Edegem, Belgium; ^6^ Department of Medical Microbiology and Infection Prevention, University Medical Center Groningen, Groningen, Netherlands

**Keywords:** acute rejection, lung transplantation, gene expression, monocytes, NanoString

## Abstract

The acute rejection (AR) diagnosis depends on transbronchial biopsy, which is semi-invasive and not easily performed**.** Our study used the Nanostring gene expression technology on PBMCs obtained from lung transplant recipients (LTRs) to search for biomarkers. We identified distinct differential gene profiles between patients with stable status (STA) and AR. Subsequently, we independently evaluated monocyte compositions in PBMCs using flow cytometry and assessed the levels of 7 chemokines in serum using Luminex. The 48 top differentially expressed genes (DEGs) were identified, utilizing a criterion of at least a 1.5-fold change between two groups, with a false discovery rate (FDR) *p*-Adj < 0.05. Of these 48 genes, the top 10 genes with the highest fold changes and significant *p*-values were selected for qPCR validation. CD68, ANXA1, ITGB, and IFI30 can be confirmed among the validated genes. A significantly lower percentage of CD14 + CD16- classical monocytes was observed in AR than in STA patients, which aligns with downregulated DEGs. Many of the DEGs were related to monocytes-macrophages and chemokines. Although these results still need to be confirmed in larger cohorts, they suggest that gene profiling of PBMC can help to identify markers related to AR in LTRs.

## Introduction

Lung transplantation (LTx) is a lifesaving option for patients with advanced lung disease for whom therapies are exhausted. Acute rejection (AR) is a life-threatening complication following graft transplantation, which is mainly caused by the cellular responses to human leukocyte antigens (HLAs) on the allograft and leads to 3.6% of deaths during the first month (1, 2). The incidence rate of AR varies around 50% in 5 years post-LTx, and approximately 28% of LTRs encountered at least one rejection episode within the initial year (3, 4). Additionally, frequent AR episodes may lead to chronic rejection, which is also one of the risk factors for developing chronic lung allograft dysfunction (CLAD) (5, 6). CLAD has been regarded as the leading cause of poor survival post-LTx (7). Early detection and diagnosis of AR could significantly influence the course of treatment and ultimately improve the chances of a successful outcome.

AR may present asymptomatic but also with clinical presentations such as fever, cough, dyspnea, and severe respiratory syndrome (4, 8, 9). Infection is a common complication post-LTx. Therefore, it may be challenging to distinguish AR from infection because of the similarity of the symptoms (10). Diagnosing AR is essential and depends on transbronchial biopsy in lung tissue (11–13). However, this is a semi-invasive procedure that is not easily performed and is accompanied by complications, such as oversedation, pneumothorax, and bleeding (14). Therefore, a noninvasive approach, measuring biomarkers in blood to diagnose AR, could be helpful.

Gene expression profiling has been performed in lung transplantation recipients (LTRs), mainly using bronchoalveolar lavage (BAL) samples (15). Previous research on genetic biomarkers was reported by Danger et al., who performed microarray analysis in peripheral blood mononuclear cells (PBMCs) and validated *POU2AF1*, *TCL1A*, and *BLK* as predictors of bronchiolitis obliterans syndrome (BOS) more than 6 months before diagnosis (16). However, similar studies for characterizing AR in LTRs have not been done so far.

In our current study, we used the NanoString® nCounter® Analysis System (NanoString Technologies, Seattle, WA, USA) with a human organ transplant Panel in PBMCs for gene profiling in LTRs with which the expression of 770 genes can be analyzed. With this technique, differential gene expression profiles have been reported in kidney (17), heart (18), lung (19), and pancreas (20) transplantation. In this study, we conducted a comparative gene expression analysis between individuals diagnosed with AR and those in stable status (STA) after LTx. The primary aim of this study was to identify potential minimally invasive biomarkers of AR to improve the clinical management of LTx patients.

## Materials and methods

### Patients and study design

PBMC samples of LTRs were selected from November 2016 until May 2022 at the University Medical Center Groningen (UMCG), the Netherlands. The local ethical committee approved the study (METc 2016/090), and all enrollments provided written informed consent. Twenty-four LTRs were selected and divided into 2 groups: (1) AR (*n* = 15), who had an occurrence of AR 1–89 months after LTx, and (2) STA (*n* = 9) were selected by age-matched and same follow-up time post-transplantation with AR patients, who were in a stable status after LTx until May 2022. On days 1 and 5 after LTx, LTRs were treated with Basiliximab. Maintenance therapy includes tacrolimus, mycophenolate mofetil (MMF), azathioprine (Aza), and prednisolone. The local pathologist assigned the AR patients following the International Society for Heart and Lung Transplantation (ISHLT) guidelines.

Blood was drawn for AR and STA patients, and serum was collected within 0–3, 3–6, 6–12, and more than 12 months after LTx. The PBMCs were isolated and stored in liquid nitrogen until use.

#### RNA isolation

PBMCs were thawed and checked for viability above 85% by trypan blue staining. Total RNA was extracted from PBMCs using the RNeasy® Kit (QIAGEN, Hilden, Germany) with the RNase-free DNase Set (QIAGEN, Hilden, Germany) manufacturers' protocols. RNA was collected in PCR tubes (AmpliStar-II PCR Tubes, Westburg) and stored at −80 °C until use. The quantity was measured with a Nanodrop 1000 system (Thermo Scientific, USA), and the quality was evaluated with the Agilent 4200 TapeStation system (Agilent Technologies, Waldbronn, Germany). Only samples with an RNA integrity number above 5.5 were used for the Nanostring analysis.

### Nanostring nCounter gene expression profiling

NanoString gene expression profiling (NanoString Technologies, Seattle, WA, USA) was performed according to suppliers instructions (21). In brief, 50 ng of RNA was incubated with capture and reported probes (Human Organ Transplant Panel, XT-CSO-HOT1-12) for 16–24 h at 65 °C. An aliquot was loaded on a SPRINT cartridge for detection and analyzed on a nCounter® SPRINT Profiler platform. The platform for subsequent gene expression analyses generated reporter code count (RCC) files.

### Nanostring nSolver and ROSALIND® differential expression gene analysis

Whole content normalization of the raw data was performed using nSolver 4.0 Analysis Software, utilizing the Code Set Content Normalization factors. Subsequently, differential expression gene analysis were performed by using nSolver 4.0 software according to the instructions (NanoString Technologies, MAN-C0019-08) and ROSALIND® (San Diego, CA) (https://rosalind.onramp.bio/). Simultaneously, the normalized data was exported in comma-separated value (CSV) format for further analyses. Principal Component Analysis (PCA) and heatmap visualization were conducted using R studio software (version 4.0.2). PCA visualization was performed using the R package ggplot2, and heatmap visualization was performed with the package heatmap. Calculation of fold changes and *p*-values for differential genes was using the fast method described in the nCounter® Advanced Analysis 2.0 User Manual. The genes with a false discovery rate (FDR) adjusted *p*-value <0.05 and fold change (FC) ≥1.5 or ≤−1.5 were considered as differentially expressed genes. The Kyoto Encyclopedia of Genes and Genomes (KEGG) pathway was evaluated using DAVID Bioinformatics Resources (NIAID/NIH, USA) by uploading the differential expressed genes. The regulated pathways were selected with an adjusted *p*-value <0.05.

### Quantitative real-time polymerase chain reaction (qRT-PCR, or qPCR)

The remaining RNA from NanoString gene expression analysis was used for qPCR validation. The reverse transcriptase (Invitrogen, USA) was used for cDNA synthesis and Taqman qPCR system (Applied Biosystems, USA) was used for the amplification according to advanced universal SYBR green mastermix instruction. We selected the following Taqman qPCR primers (Taqman, USA): *CD68* (Hs00154355_m1), *ANXA1* (Hs00167549_m1), *ITGB2* (Hs00164957_m1), *IFI30* (Hs00173838_m1), *LTBR* (Hs01101194_m1), *CCR1* (Hs00174298_m1), *FKBP1A* (Hs00356621_g1), *MYD88* (Hs00182082_m1), *TNFRSF1A* (Hs01042313_m1), *TNFRSF1B* (Hs00961750_m1). Besides, *RPLP0* (Hs99999902_m1), *GAPDH* (Hs99999905_m1), and *B2M* (Hs99999907_m1) were used as reference gene primers for normalization (22). The household genes showed no significant differences between the AR and STA samples. The data was analyzed using QuantStudioTM Real-Time PCR System (USA). The 2^−Δ*Ct*^ method was used to calculate the relative expression.

### Luminex for chemokines measurements

Levels of the chemokines CCL2, CX3CL1, CXCL10, CXCL16, CXCL6, CXCL8, and CXCL1 in serum were measured by using the Luminex Discovery Assay (Biotechne, R&D systems, USA) according to the manufacturer's instructions. Subsequently, the assay results were obtained using a Luminex Magpix instrument, and the data were analyzed using Xponent 4.2 software (Bio-Techne, R&D Systems, USA).

### Flow cytometry

For the monocytes analysis, PBMCs were thawed and stained with antibodies specific for HLA-DR (clone: TU36, BD), CD14 (clone: rmC5-3, BD), CD16 (clone: B73.1, BD). The cells were measured, and data acquisition was performed using the Symphony flow cytometer (BD). 500.000 events per sample were recorded. The data was processed using the analysis software Kaluza (Beckman Coulter, USA) with a gating strategy as described (23) in the Supplementary Figure 1. To eliminate potentially contaminating lymphocytes from the monocyte gate, negative cells for both HLA-DR and CD14 were excluded.

### Statistical analysis

Continuous data are presented as median and range. Differences between the two groups were tested using Mann–Whitney *U* test. Additionally, differences between the clinical parameters among STA and AR groups were analyzed using a Chi-square test. Data analysis was performed using IBM SPSS statistics version 23.0, and figures were created with GraphPad Prism version 9.0. Statistical significance was defined as *p*-value <0.05 (two-tailed).

## Results

### Baseline characteristics

**Figure 1:**
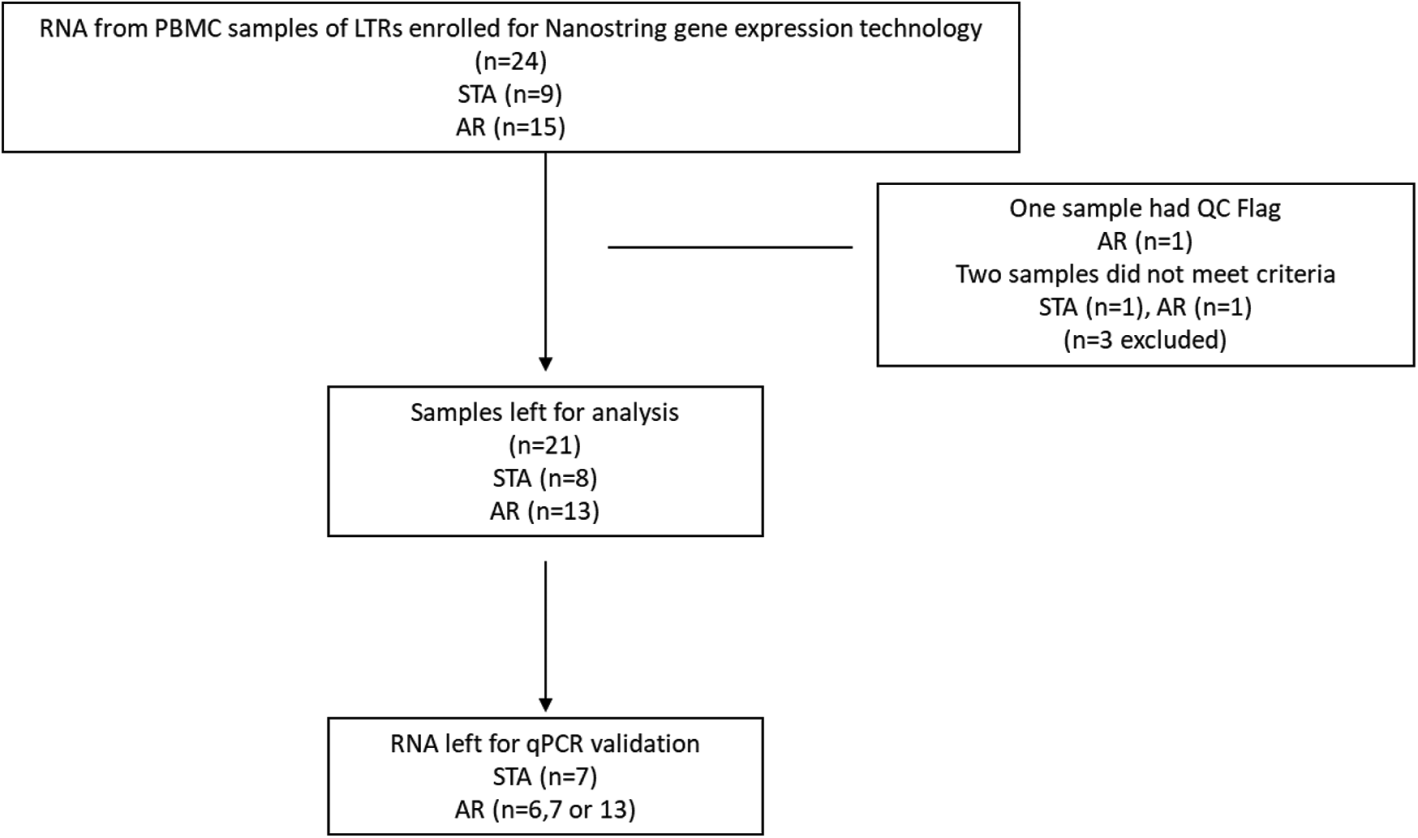
Flow chart of participants enrolled for nanostring gene expression.

Initially, RNA samples from 24 participants were selected (Figure 1). Subsequently, 21 samples meeting the quality criteria were categorized into AR (*n* = 13) and STA (*n* = 8) patients. Table 1 provides an overview of the baseline characteristics of these 21 participants, which are included for further analysis. While age and sex differences between the two groups were not statistically significant, it is noteworthy that the AR group had slightly younger patients and a higher proportion of males than the STA group.

**Table 1:** Biological characteristics of participants.

Characteristics	AR (*n* = 13)	STA (*n* = 8)	*p*-value
Age at Tx (range)	53 (15–65)	64 (55–69)	ns
Sex, *N* (%)			
Male	9 (69.2)	4 (50)	ns
Female	3 (30.8)	4 (50)	
Time of AR after LTx, months (range)	14.6 (1–89)	—	
PBMC isolation after AR, days (range)	34 (1–83)	—	
PBMC isolation after LTx, months (range)	15.7 (1–89)	4.0 (1.0–8.4)	ns
Underlying disease *N* (%)			
COPD/Emphysema	8 (61.5)	4 (50)	ns
Alpha-1-Antitrypsin Deficiency	4 (30.8)	1 (12.5)	
Cystic fibrosis/bronchiectasis	1 (7.7)	0 (0)	
Pulmonary Fibrosis/Interstitial lung disease (ILD)	0 (0)	3 (37.5)	
Immunosuppression (%)			
Tac-MMF-Pred	13 (100)	7 (87.5)	ns
Tac-MMF-Pred change to Tac-Aza-Pred	0 (0)	1 (12.5)	
Donor(D)/Recipient(R) CMV serostatus, *N* (%)			
D+/R+	5 (38.5)	1 (12.5)	ns
D+/R−	1 (7.7)	3 (37.5)	
D−/R+	3 (23.1)	3 (37.5)	
D−/R−	4 (30.7)	1 (12.5)	
Primary graft dysfunction (PGD) at 48 h			
Level 0	8 (61.5)	—	—
Level 1	3 (23.1)	—	—
Level 2	0 (0)	—	—
Level 3	2 (15.4)	—	—

Tac, tacrolimus; MMF, mycophenolate-mofetil; pred, prednisolone; Aza, azathioprine.

*P*-values results are based on chi-square and Mann–Whitney *U* test analysis between STA and AR groups.

AR manifested at a median of 14.6 months (1–89 months) post-LTx, with PBMC sampling occurring at a median of 34 days (ranging from 1 to 83 days) after AR onset. Of note, 2 of 13 AR patients had AR more than one year after LTx, after they had received a dose reduction of the medication due to toxicity. PBMCs from AR and STA patients were isolated 15.7 (1–89) months and 4.0 (1.0–8.4) months after LTx, respectively.

COPD/emphysema was the leading cause of LTx in 61.5% of STA patients and 50% of AR patients. Seven of 8 STA and all AR patients received immunosuppressive treatment consisting of basiliximab induction (anti-CD25; 2 doses of 20 mg) followed by Tacrolimus (Tac), MMF, and prednisolone (Pred) maintenance therapy. MMF was switched to Azathioprine if side effects happened. A single patient in the STA group was initially treated with Tac-MMF-Pred and subsequently transitioned to a regimen of Tac-Azathioprine (Aza)-Pred. Treatment of AR patients consisted of pulse methylprednisolone (1 g intravenously for 3 days).

### Identification of differential expression gene signatures

**Figure 2:**
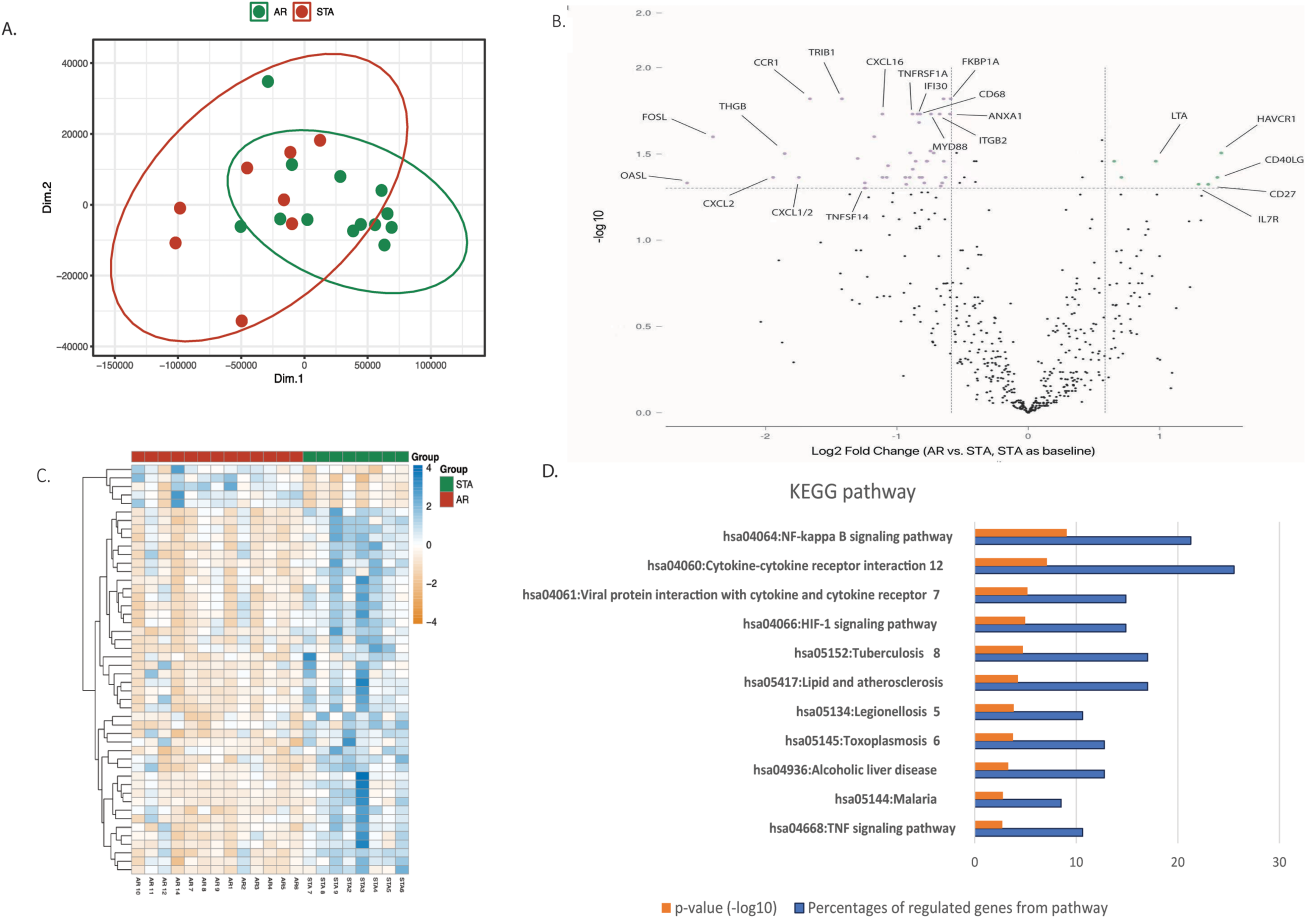
Differential expression genes. **(A)** Principal Component Analysis (PCA) comparing the expression profiles of 770 genes in the AR (green dots) and STA (red dots) groups. **(B)** The volcano plot displays transcripts that are upregulated (green dots) and downregulated (pink dots) genes with a false discovery rate (FDR) adjusted *p*-value ≤0.05 and a log2 fold change >1.5 in the AR group compared to the STA group (STA used as the baseline). **(C)** Heatmap illustrating the 48 differential expression of genes distinguishing the AR (green) and STA (red) groups. The *p*-value was calculated by independent *t*-test between PC1 and PC2 using R software. **(D)** KEGG pathway descriptors. The orange column represents the numeric -log10 *p*-value, blue column represents the percentage of involved genes of all 48 differential expression genes. The pathways were selected by adjusted *p*-value <0.05 of KEGG pathways evaluated in DAVID Bioinformatics Resources system.

NanoString analysis was performed to identify differentially expressed genes between AR and STA patients. After normalization of the data, PCA analysis of the 770 analyzed genes showed significant differences in expression between the STA and AR groups (Figure 2A, *p*-value = 0.009). A volcano plot (Figure 2B) demonstrated increased expression for 7 genes and decreased expression for 41 genes by at least a 1.5-fold change in the AR group as compared to the STA group at an FDR-adjusted *p*-value < 0.05 (Table S.1). The normalized counts of these highly differentially expressed genes were visualized in a heatmap (Figure 2C). Eleven pathways with a significant adjusted *p*-value <0.05 in KEGG pathway analysis were observed (Figure 2D). Of note was the NF-kappa B signaling pathway (*p* < 0.001), which involved 11 out of 48 differential expression genes. Of the 11 genes, 9 were downregulated (*CXCL2*, *TNFSF14*, *NFIL13*, *BCL2A1*, *CD14*, *TNFRSF1A*, *TLR4*, *LTBR*, *MYD88*) and 2 were upregulated (*CD40LG*, *LTA*).

### Validation of differentially expressed genes by qPCR

**Figure 3:**
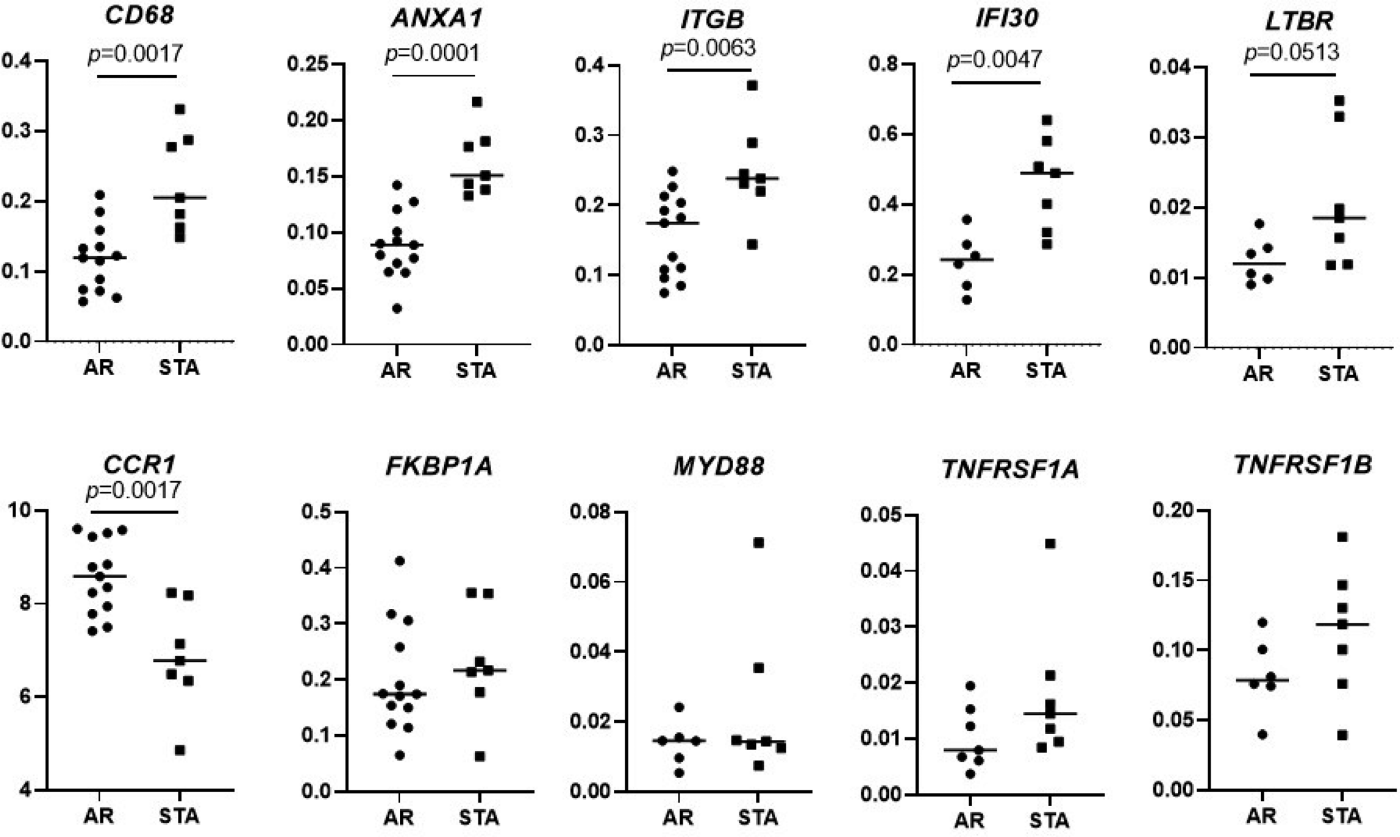
Validation of differential expressed genes. Validation of differential expression genes of *CD68*, *ANXA1*, *ITGB*, *IFI30*, *LTBR*, *CCR1*, *FKBP1A*, *MYD88*, *TNFRSF1A*, *TNFRSF1B* by quantitative PCR in the same patients set for Nanostring gene expression. Differences between the two groups were tested using the Mann–Whitney *U* test.

The top 10 differentially expressed genes (highest fold changes with significant *p*-values) between the STA and AR groups were chosen for qPCR validation: *CD68*, *ANXA1*, *ITGB*, *IFI30*, *LTBR*, *CCR1*, *FKBP1A*, *MYD88*, *TNFRSF1A*, *TNFRSF1B* were downregulated in AR compared STA group. Expression of *CD68* (*p* = 0.0017), *ANXA1* (*p* = 0.0001), *ITGB* (*p* = 0.0063), and *IFI30* (*p* = 0.0047) was significantly decreased in the AR patients compared to the STA patients (Figure 3). Also, *FKBP1A*, *TNFRSF1A*, and *TNFRSF1B* were decreased in AR, but no significance was reached. In contrast, expression of *CCR1* was upregulated in AR compared to STA patients (*p* = 0.0017).

### Independent evaluation of chemokines and monocytes

**Figure 4:**
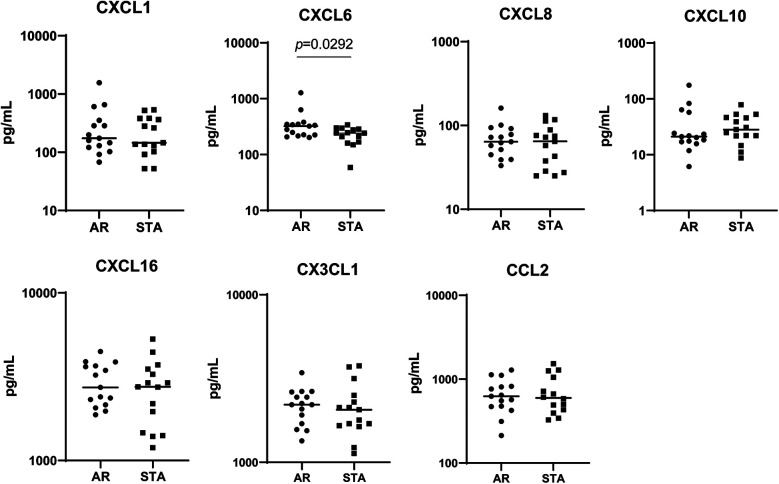
Analysis of chemokines in serum. An independent validation of extended Chemokines (CXCL1, CXCL6, CXCL8, CXCL10, CXCL16, CX3CL1, and CCL2) using Luminex for Comparing AR and STA Patients.

As shown in Supplementary Table S1, we identified differential expression of several chemokines, specifically CXCL16 and CXCL1. Subsequently, we performed an independent evaluation study with 30 serum samples from LTRs, comprising 15 STA and 15 AR patients, matched for age and post-LTx time (as detailed in Supplementary Table S2). We extended the analysis to include other relevant chemokines: CCL2, CX3CL2, CXCL10, CXCL8, and CXCL6. However, the lower expression of CXCL16 and CXCL1 could not be confirmed at the protein level. Surprisingly, CXCL6 levels were significantly higher in AR patients compared to STA patients (Figure 4, *p* = 0.0292).

**Figure 5:**
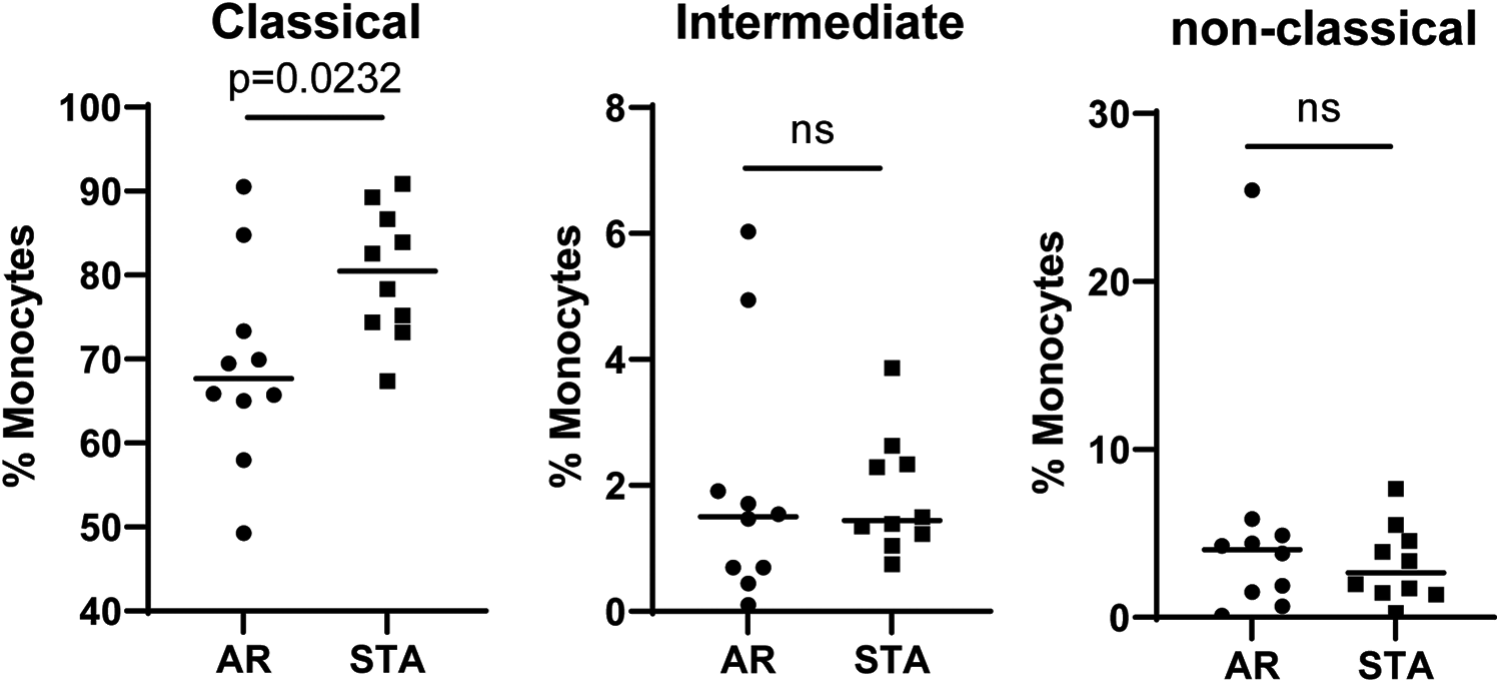
An independent validation of monocytes in PBMCs. Validation of CD14 + CD16− classical monocytes, CD14 + CD16 + intermediate monocytes, and CD14 + CD16− classical monocytes by flow cytometry comparing AR and STA patients.

Because CD14 and CD68 are in the list of differentially expressed genes highly expressed by monocyte lineage cells (24, 25), we further assessed frequencies of monocytes in PBMCs of AR and STA groups by flow cytometry. Among the patients selected for chemokine analysis, 10 STA and 10 AR patients had PBMC samples available for monocyte analysis using flow cytometry. Monocytes were categorized as classical (CD14+, CD16-), intermediate (CD14+, CD16+), and non-classical (CD14−, CD16+) monocytes. As demonstrated in Figure 5, there was a significantly reduced frequency of CD14 + CD16− classical monocytes (*p* = 0.0232) in the AR group compared to STA.

## Discussion

In our study, we profiled PBMCs to identify AR-associated genes in LTRs. We validated four genes (*CD68*, *ANXA1*, *ITGB*, *IFI30*) statistically differentiating AR and STA patients with qPCR. Additionally, our cellular analysis of monocytes highlighted significant distinctions of reduced classical monocyte CD14 + CD16- frequencies in AR patients and the upregulation of CXCL6 levels compared to STA patients.

We observed a significantly reduced expression of CD68 in AR patients in gene expression analysis. CD68, the human homolog of macrosialin, is commonly regarded as a lineage marker for human monocytes and macrophages (24). In PBMCs, CD68 is not expressed on monocytes except when they differentiate towards macrophages. Classification of monocytes in PBMCs can be further analyzed by expression of CD14 (LPS co-receptor) and CD16 (Fc*γ* receptor III) that define three phenotypically and functionally distinct human monocyte subsets: CD14 + CD16− (classical), CD14 + CD16+ (intermediate), and CD14-CD16+ (non-classical) monocytes. Until now, no studies have reported the relation between monocyte compositions and AR after LTx. Our results indicate that the CD14 + CD16− classical monocytes percentage was lower in AR than in STA patients. In previous studies, the experimental depletion of monocytes in murine heart and kidney transplantation models demonstrated a reduction in T cell-mediated rejection (26). Additionally, in a study of murine LTx, CD4 T cells from recipients lacking circulating classical monocytes were shielded from allorecognition, but AR in the allografts persisted (27). This evidence suggests a potential inter-connection between lower levels of monocytes and AR post-transplantation.

Furthermore, the classical monocytes can differentiate into macrophages in tissue (28), which can occur during inflammatory conditions (29). The reduced classical monocyte percentage in PBMCs of AR patients implies there may be more monocyte-to-macrophage differentiation in AR development, resulting in enhanced migration of these monocytes to the tissue and thereby reduced levels of these monocytes. This suggests that AR patients may have a heightened capacity for monocyte transformation into tissue-resident macrophages, emphasizing the importance of inflammation in rejection. Due to the lack of lung tissues from AR patients, we could not further validate macrophages in tissue. Nevertheless, it has been demonstrated that by employing immunohistochemical double staining of CD68 and CD31, the accuracy of diagnosing antibody-mediated rejection in heart transplantation can be enhanced (30). Also, a study using a rat model of LTx found that CD68 + macrophages were the predominant cell type observed during AR through immunohistochemistry (31).

What's more, a study utilizing PROMAD identifies myeloid cells as the origin of acute rejection markers, emphasizing their critical role and immunologic memory in modulating transplant rejection, supported by evidence of CD16 + monocyte/macrophage prevalence in rejecting heart transplants and resident macrophages shaping lung allograft immune responses (32).

Macrophages are crucial in acute antibody-mediated rejection (AMR) and cellular rejection (ACR). Their presence in rejected allograft tissue is related to poorer graft function and survival (33). Notably, corticosteroids in immunosuppressive treatments may alter monocyte subpopulation distribution. Still, therapies involving MMF, calcineurin inhibitors, and mammalian target of rapamycin inhibitors (mTOR) have not shown significant effects in this regard (34).

Furthermore, we cross-referenced our differentially expressed genes (DEG) data with related organ transplantation studies. *CD14*, *IFI30*, *TNFRSF1B*, *CD27,* and *ITGB2* emerged as significant DEG associated with AR, aligning with our findings (35). To our knowledge, no subsequent validation studies have been conducted on these genes related to AR. Of note, *ANXA1*, a member of the Annexins (ANXs) family, known for its calcium and phospholipid binding properties, has been related to lung injury and inflammation (36). The lower expression of *ANXA1* in AR patients compared to STA patients may be attributed to its anti-inflammatory role. *IFI30* (GILT), a lysosomal thiol reductase, plays a role in catalyzing the reduction of disulfide bonds within protein antigens. This process aids antigen-presenting cells (APCs) in effectively presenting antigens to T cells while downregulating regulatory T cells (Tregs) (37, 38). Our findings of lower expression of IFI30 in AR patients might signify a potential reduction in immune tolerance. In a differential gene expression study involving mucosal biopsies of rejection and stable patients in LTx, *ITGB2* was identified as a hub gene with theoretical significance in the inhibition of rejection, as revealed in drug-gene interaction analysis (39). Our findings align with this, suggesting that *ITGB2* could also be a potential biomarker.

We also noticed the potential involvement of chemokines, particularly CXCL6, in AR. This corresponds with findings in kidney transplantation, where CXCL6 levels were higher in the AR group compared to the non-AR group (40). In a liver transplantation study, increased secretion of CXCL1 and CXCL2 was observed, which can boost the functional activity of the mononuclear phagocyte system and possibly contribute to processes associated with liver rejection (41).

Our KEGG analysis implied the significant involvement of the NF-kappa B signaling pathway during AR. Several organ transplantation studies have shown that NF-kappa B signaling activation can lead to rejection, and inhibiting NF-kappa B in specific cell types may enhance graft survival in solid organ allograft rejection (42–44). After transplantation, NF-kappa B activation occurs shortly after ischemia/reperfusion and is later reactivated within infiltrating cells during AR. The literature underscores the role of NF-kappa B activation in the detrimental effects of ischemia/reperfusion, the survival of activated T cells, differentiation of various effector T cell types, memory T cell formation, and dendritic cell maturation. Notably, NF-kappa B activation may counteract the peripheral development of Tregs, which can suppress the immune response against allografts and prolong graft survival (44, 45). Furthermore, the activation of NF-kappa B signaling plays a pivotal role in generating natural Tregs, underscoring its significance in the context of solid organ allograft rejection (46).

In a kidney transplantation study, the effects of sample timing and treatment on gene expression were shown in early AR (47). This emphasizes the importance of moment when assessing gene expression during AR. Future research should study biomarkers at different time points post-LTx to identify essential genes in the process of AR.

The main limitation of our study is the relatively small cohort size. Additionally, not all samples, especially from AR patients, were available at every time point due to patient no-shows and participation in other clinical studies, contributing to the unavailability of some samples at specific time points. Further studies were recommended to validate these with simplified samples.

In summary, by using minimally invasive samples, we identified significant differentially expressed genes. *CD68*, *ANXA1*, *ITGB*, and *IFI30* were successfully validated, suggesting involvement in AR-related immune response and inflammation. Further research should reveal their precise roles as potential diagnostic or prognostic markers for AR. Additionally, our results shed light on the possible role of monocyte compositions and the NF-kappa B signaling pathway in AR development. These findings hold promise for aiding clinicians in identifying novel biomarkers to enhance the accuracy of diagnosing AR.

## Data Availability

The original contributions presented in the study are included in the article/Supplementary Material, further inquiries can be directed to the corresponding author.
