# Supplementary figures and images for: Gene expression profiling in PBMCs for acute rejection in lung transplant recipients reveals myeloid responses
Source: Front Transplant. 2024 Dec 18;3:1508419. doi: 10.3389/frtra.2024.1508419 (PMC11688322; doi:10.3389/frtra.2024.1508419)

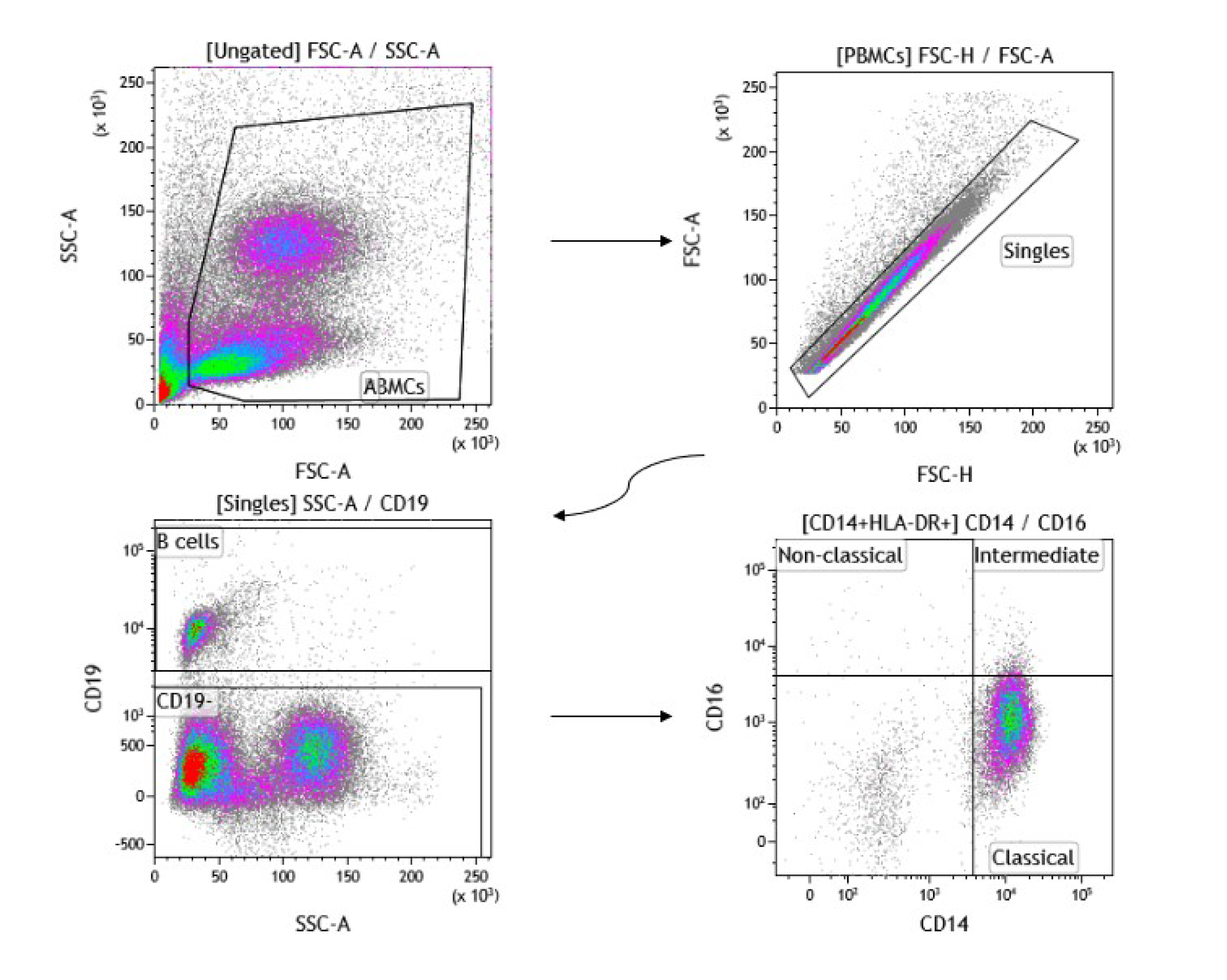

Supplement: Supplementary file 1 [file Image1.tif]
