# Supplementary material for: Gene expression profiling in PBMCs for acute rejection in lung transplant recipients reveals myeloid responses
Source: Front Transplant. 2024 Dec 18;3:1508419. doi: 10.3389/frtra.2024.1508419 (PMC11688322; doi:10.3389/frtra.2024.1508419)
Supplement: Supplementary file 2 [file Table1.docx]

Supplementary Table.1 Top 48 differentially expressed genes in AR compared to STA.

| **Name** | **Description** | **Fold Change** | **Log Fold Change** | **p-Value** | **p-Adj** | **Average Log2 Expression** |
| --- | --- | --- | --- | --- | --- | --- |
| ***CCR1*** | chemokine (C-C motif) receptor 1 | -3.16538 | -1.66238 | 9.26E-05 | 0.01515 | 8.3847 |
| ***FKBP1A*** | FK506 binding protein 1A, 12kDa | -1.5066 | -0.591299 | 3.42E-05 | 0.01515 | 7.6378 |
| ***GAPDH*** | glyceraldehyde-3-phosphate dehydrogenase | -1.5642 | -0.645425 | 9.64E-05 | 0.01515 | 11.0567 |
| ***TRIB1*** | tribbles pseudokinase 1 | -2.67341 | -1.41868 | 0.000116 | 0.01515 | 9.1234 |
| ***ANXA1*** | annexin A1 | -1.51134 | -0.595826 | 0.000211 | 0.01856 | 13.2574 |
| ***CD68*** | CD68 molecule | -1.79343 | -0.842718 | 0.000248 | 0.01856 | 8.875 |
| ***ITGB2*** | integrin, beta 2 (complement component 3 receptor 3 and 4 subunit) | -1.59637 | -0.674795 | 0.00036 | 0.01856 | 9.2595 |
| ***MYD88*** | myeloid differentiation primary response 88 | -1.67141 | -0.741064 | 0.00039 | 0.01856 | 9.971 |
| ***TNFRSF1A*** | tumor necrosis factor receptor superfamily, member 1A | -1.84147 | -0.880857 | 0.000288 | 0.01856 | 8.2736 |
| ***IFI30*** | interferon, gamma-inducible protein 30 | -1.76946 | -0.823313 | 0.000322 | 0.01856 | 9.6903 |
| ***CXCL16*** | chemokine (C-X-C motif) ligand 16 | -2.15983 | -1.11092 | 0.00034 | 0.01856 | 6.8896 |
| ***LTBR*** | lymphotoxin beta receptor (TNFR superfamily, member 3) | -1.7795 | -0.83147 | 0.000476 | 0.02078 | 7.1119 |
| ***PLAUR*** | plasminogen activator, urokinase receptor | -2.25408 | -1.17254 | 0.000623 | 0.0251 | 8.3686 |
| ***FOSL1*** | FOS-like antigen 1 | -5.28125 | -2.40088 | 0.000672 | 0.02516 | 4.4053 |
| ***TNFRSF1B*** | tumor necrosis factor receptor superfamily, member 1B | -1.67569 | -0.744758 | 0.000928 | 0.03039 | 8.6995 |
| ***CD14*** | CD14 molecule | -1.8647 | -0.898941 | 0.001126 | 0.03119 | 10.6794 |
| ***IFNGR2*** | interferon gamma receptor 2 (interferon gamma transducer 1) | -1.64752 | -0.720293 | 0.001035 | 0.03119 | 9.834 |
| ***HAVCR1*** | hepatitis A virus cellular receptor 1 | 2.76924 | 1.46949 | 0.00118 | 0.03119 | 3.5639 |
| ***THBD*** | thrombomodulin | -3.61822 | -1.85528 | 0.001261 | 0.03146 | 8.1144 |
| ***SERTAD1*** | SERTA domain containing 1 | -2.46045 | -1.29892 | 0.00141 | 0.03359 | 5.78 |
| ***VCAN*** | versican | -1.81259 | -0.858056 | 0.001786 | 0.03483 | 9.0932 |
| ***FCER1G*** | Fc fragment of IgE, high affinity I, receptor for; gamma polypeptide | -1.70924 | -0.773356 | 0.001533 | 0.03483 | 10.7972 |
| ***LTA*** | lymphotoxin alpha | 1.95998 | 0.970841 | 0.002002 | 0.03483 | 5.1307 |
| ***RPS6KB1*** | ribosomal protein S6 kinase, 70kDa, polypeptide 1 | 1.57365 | 0.654113 | 0.001946 | 0.03483 | 8.3729 |
| ***TIMP1*** | TIMP metallopeptidase inhibitor 1 | -1.7058 | -0.770448 | 0.002053 | 0.03483 | 10.2983 |
| ***LILRB2*** | leukocyte immunoglobulin-like receptor, subfamily B (with TM and ITIM domains), member 2 | -1.56219 | -0.643573 | 0.001915 | 0.03483 | 8.184 |
| ***TREM1*** | triggering receptor expressed on myeloid cells 1 | -2.09628 | -1.06783 | 0.002259 | 0.03699 | 9.4275 |
| ***TLR4*** | toll-like receptor 4 | -1.83981 | -0.879557 | 0.002441 | 0.03877 | 9.8335 |
| ***CD40LG*** | CD40 ligand | 2.71261 | 1.43968 | 0.003671 | 0.04322 | 6.0444 |
| ***DNMT3A*** | DNA (cytosine-5-)-methyltransferase 3 alpha | 1.63534 | 0.709592 | 0.003305 | 0.04322 | 7.0481 |
| ***CXCL2*** | chemokine (C-X-C motif) ligand 2 | -3.84475 | -1.94289 | 0.002936 | 0.04322 | 8.8498 |
| ***IMPDH1*** | IMP (inosine 5'-monophosphate) dehydrogenase 1 | -1.54734 | -0.629789 | 0.003356 | 0.04322 | 7.9388 |
| ***LDLR*** | low density lipoprotein receptor | -2.15864 | -1.11012 | 0.003243 | 0.04322 | 6.2866 |
| ***NFIL3*** | nuclear factor, interleukin 3 regulated | -1.91274 | -0.935641 | 0.003507 | 0.04322 | 9.371 |
| ***P2RX4*** | purinergic receptor P2X, ligand gated ion channel, 4 | -1.77577 | -0.828447 | 0.003504 | 0.04322 | 4.9442 |
| ***S100A8*** | S100 calcium binding protein A8 | -2.11064 | -1.07768 | 0.003262 | 0.04322 | 14.7209 |
| ***NOD2*** | nucleotide-binding oligomerization domain containing 2 | -1.75175 | -0.808794 | 0.002856 | 0.04322 | 6.3611 |
| ***MS4A6A*** | membrane-spanning 4-domains, subfamily A, member 6A | -1.86926 | -0.902468 | 0.003752 | 0.04322 | 9.1591 |
| ***CXCL1/2*** | peptide belonging to the CXC chemokine family | -3.3556 | -1.74657 | 0.002986 | 0.04322 | 8.5396 |
| ***AHR*** | aryl hydrocarbon receptor | -1.73085 | -0.791483 | 0.004538 | 0.04655 | 9.4997 |
| ***C3AR1*** | complement component 3a receptor 1 | -2.36904 | -1.2443 | 0.004578 | 0.04655 | 7.3466 |
| ***HK2*** | hexokinase 2 | -1.57491 | -0.655269 | 0.004618 | 0.04655 | 7.6204 |
| ***OASL*** | 2'-5'-oligoadenylate synthetase-like | -6.0543 | -2.59796 | 0.00462 | 0.04655 | 3.8331 |
| ***IL7R*** | interleukin 7 receptor | 2.45668 | 1.29671 | 0.004793 | 0.04739 | 10.6818 |
| ***BCL2A1*** | BCL2-related protein A1 | -1.90389 | -0.928949 | 0.004946 | 0.04744 | 9.9122 |
| ***CD27*** | CD27 molecule | 2.58573 | 1.37057 | 0.004979 | 0.04744 | 7.356 |
| ***RXRA*** | retinoid X receptor, alpha | -1.58671 | -0.666041 | 0.005188 | 0.04854 | 8.7209 |
| ***TNFSF14*** | tumor necrosis factor (ligand) superfamily, member 14 | -2.36685 | -1.24297 | 0.005424 | 0.04986 | 4.6604 |

Supplementary Table 2. Biological characteristics of participants

| **Characteristics** | **AR (n=15)** | **STA (n=15)** | **p-value** |
| --- | --- | --- | --- |
| Age at Tx (range) | 59 (30-75) | 62 (31-73) | ns |
| Sex, N % | | | |
| Male | 7 (46.7) | 6 (40) | ns |
| Time of AR after LTx, days (range) | 62 (9-338) | - |  |
| PBMC isolation after AR, days (range) | 32 (2-79) | - |  |
| PBMC isolation after LTx , months (range) | 178 (30-365) | 89 (30-364) | ns |
| Underlying disease. N (%) | | | |
| COPD/Emphysema | 11 (73.3) | 12 (80) | ns |
| Alpha-1-Antitrypsin Deficiency | 2 (13.3) | 0 (0) |  |
| Cystic fibrosis/bronchiectasis | 2 (13.3) | 1 (6.7) |  |
| Pulmonary Fibrosis/Interstitial lung disease (ILD) | 0 (0) | 2 (13.3) |  |
| Immunosuppression | | | |
| Tac-MMF-Pred | 14 (93.3) | 14 (93.3) | ns |
| Tac-MMF-Pred change to Tac-Aza-Pred | 1 (6.7) | 1 (6.7) |  |

Tac: Tacrolimus; MMF: Mycophenolate-mofetil; pred: prednisolone; Aza: Azathioprine.

P-values results are based on chi-square and Mann Whitney U test analysis between STA and AR groups.
